# Anti-IgE monoclonal antibody therapy for the treatment of chronic rhinosinusitis: a systematic review

**DOI:** 10.1186/s13643-015-0157-5

**Published:** 2015-11-18

**Authors:** Chris J. Hong, Adrian C. Tsang, Jason G. Quinn, James P. Bonaparte, Adrienne Stevens, Shaun J. Kilty

**Affiliations:** Faculty of Medicine, University of Ottawa, Ottawa, ON Canada; Department of Pathology and Laboratory Medicine, Dalhousie University, Halifax, NS Canada; Department of Otolaryngology-Head and Neck Surgery, University of Ottawa, The Ottawa Hospital, 737 Parkdale Ave., Room 459, Ottawa, Ontario K1Y 1J8 Canada; Center for Practice Changing Research, Ottawa Hospital Research Institute (OHRI), Ottawa, ON Canada; Department of Clinical Epidemiology, University of Ottawa, Ottawa, ON Canada

**Keywords:** Anti-IgE monoclonal antibody, Asthma, Chronic rhinosinusitis, Nasal polyps

## Abstract

**Background:**

Several options are available for the treatment of chronic rhinosinusitis (CRS), but disease control remains elusive for many patients. Recently, literature has emerged describing anti-IgE monoclonal antibody as a potential therapy for CRS. However, its effectiveness and safety are not well known. The purpose of this systematic review was to assess the effectiveness and safety of anti-IgE therapy and to identify evidence gaps that will guide future research for the management of CRS.

**Methods:**

Methodology was registered with PROSPERO (No. CRD42014007600). A comprehensive search was performed of standard bibliographic databases, Google Scholar, and clinical trials registries. Only randomized controlled trials assessing anti-IgE therapy in adult patients for the treatment of CRS were included. Two independent reviewers extracted data using a pre-defined extraction form and performed quality assessment using the Cochrane risk of bias tool and the GRADE framework.

**Results:**

Two studies met our inclusion criteria. When comparing anti-IgE therapy to placebo, there was a significant difference in Lund-McKay score (*p* = 0.04) while no difference was seen for percent opacification on computed tomography (CT). At 16 weeks, treatment led to a decrease in clinical polyp score. No significant difference was seen with regard to quality of life (Total Nasal Symptom Severity (TNSS), *p* < 0.21; Sinonasal Outcome Test 20 (SNOT-20), *p* < 0.60), and no serious complications were reported in either trial. Based on the quality assessment, studies were deemed to be of moderate risk of bias and a low overall quality of evidence.

**Conclusions:**

There is currently insufficient evidence to determine the effectiveness of anti-IgE monoclonal antibody therapy for the treatment of CRS.

**Electronic supplementary material:**

The online version of this article (doi:10.1186/s13643-015-0157-5) contains supplementary material, which is available to authorized users.

## Background

Chronic rhinosinusitis (CRS) is a condition characterized by persistent inflammation of the paranasal sinus mucosa with concomitant bacterial colonization. It is a disease of high prevalence, especially in women and older adults [1]. It affects 14.2 % of the adult US population and accounts for between 18 and 22 million annual office visits [2–4]. In Canada, CRS affects about 5 % of adults and accounts for close to one million prescriptions annually [1]. The symptoms of CRS significantly lower the physical and psychological well-being of patients, which directly leads to decreased patient quality of life [5–8].

Recent advances in understanding of the pathophysiology of CRS have improved management paradigms, which have resulted in improved quality of life for CRS patients [8]. Several medical and surgical alternatives are available, ranging from corticosteroids and antibiotics to functional endoscopic sinus surgery. Despite improved outcomes for CRS patients, these treatment strategies are limited since they focus on symptom relief and inflammatory reduction rather than causal abatements. Consequently, disease control remains elusive for many patients, particularly for those with nasal polyposis and comorbid asthma [9]. Current investigations have shifted focus to target the genesis of dysregulated mucosal immune and barrier responses and the resultant chronic sinonasal inflammation states [10, 11]. Based on the proposed role of eosinophils and Th_2_ cytokines in CRS, IgE—a key inflammatory mediator—has been implicated in the pathophysiology of CRS of some patients [12]. Similarly, literature has emerged that describes anti-IgE monoclonal antibody (omalizumab) as a potential injection therapy for CRS [13–17].

There are several studies that examine the role of anti-IgE monoclonal antibody in the management of CRS [13–15, 18–20], but its effectiveness and safety are not well known. The purpose of this systematic review was twofold: (1) to assess the effectiveness and safety of anti-IgE monoclonal antibody therapy for the treatment of adult patients with CRS and (2) to identify evidence gaps that will guide future research on anti-IgE monoclonal antibody therapy for the management of CRS.

## Methods

We undertook a systematic review based on an a priori protocol that was registered with PROSPERO (No. CRD42014007600) [21]. This systematic review has been reported according to the Preferred Reporting Items for Systematic reviews and Meta-Analyses (PRISMA) statement [22] (Additional file 1).

### Search strategy and selection process

A comprehensive search was performed of standard bibliographic databases (MEDLINE, EMBASE, CINAHL, Web of Science, and The Cochrane Library). Google Scholar was searched as a source of gray literature. A manual search of relevant journals and conference proceedings was also performed. The reference lists of included articles as well as relevant review articles were screened to identify any relevant studies that were not previously identified. ClinicalTrials.gov, International Clinical Trials Registry Platform (ICTRP), and EU Clinical Trials Registry were also searched to identify any ongoing or completed trials. All studies written in English published between January 1970 and April 2014 were included. The following key words and Mesh controlled vocabulary terms were used in varying combinations: *antibodies*, *monoclonal*; *antibodies*, *anti-idiotypic*; *antibodies*, *monoclonal*, *humanized*; *sinusitis*; *rhinitis*; and *nasal polyps* (Appendix 1). The MEDLINE search was adapted to the other databases.

Titles and abstracts of the retrieved articles were then screened for their potential relevance by a single reviewer (SK). The full-text versions of potentially relevant articles were obtained and assessed using a pre-defined eligibility form by the same reviewer. Only randomized controlled trials (RCTs) assessing anti-IgE monoclonal antibody therapy in adult (>18) patients for the treatment of CRS were included.

### Eligibility criteria

- Population: adult patients (>18) with CRS, even if the condition was poorly defined.
- Intervention and comparison: studies comparing anti-IgE monoclonal antibody therapy with placebo or another therapy, given for at least 16 weeks; anti-IgE in combination with other therapies or as an adjuvant therapy was not assessed here.
- Outcomes (not used for selection of studies): outcomes were collected for any period of follow-up.
- Primary outcomes: change in computed tomography (CT) score, change in clinical polyp score, and change in quality of life.
- Secondary outcomes: change in cellular inflammation, change in nasal airflow, change in olfaction, adverse events, change in systemic IgE levels, and change in spirometric results.
- Study design: RCTs.
- Timing: studies published or reported as of 1970 were included (1970 was the earliest available year on standard bibliographic databases).
- Language: studies written in the English language were included.

### Data extraction

Two independent reviewers (JQ and JB) read full-text reports and extracted data using a pre-defined extraction form. Data were extracted on the following: title, first author, year of publication, general study and patient characteristics, study methods, and outcome definitions and data. Refer to Table 1 for details on data extraction elements. Discrepancies were settled by consensus and discussion amongst the reviewers.

**Table 1:** Data extraction elements

General data extraction elements
• Study number
• First author
• Publication year
• Country
• Funding source
• Study design
• Polyp staging score used
• Inclusion criteria
• Exclusion criteria
• Subjects (total (*n*), men (*n*), women (*n*))
• Age (mean, median, range)
• Number excluded
• Study duration
Data extraction elements for the treatment (anti-IgE) and placebo arms
• Subjects (total *n*, subjects with sinusitis and polyps (*n*), subjects with sinusitis without polyps (*n*), men (*n*), women (*n*), % subjects with asthma, % subjects with history of immunotherapy, subjects with allergy (*n*, %), number of dropouts/withdrawals from study)
• Age (mean, median, range)
• BMI (mean, median, range)
• Disease severity
• Concurrent medications
• Comorbid conditions
• Dosing regimen, dose amount, duration, frequency
• Pre-CT score ± SD, post-CT score ± SD, length of follow-up, sample size
• Pre-clinical polyp score ± SD, post-clinical polyp score ± SD, length of follow-up, sample size
• Pre-quality of life instrument (SF-36, AQLQ, RSOM-31, TNSS, SNOT-20) ± SD, post-quality of life instrument (SF-36, AQLQ, RSOM-31, TNSS, SNOT-20) ± SD, length of follow-up, sample size
• Pre-cellular inflammation (eosinophil count) ± SD, post-cellular inflammation (eosinophil count) ± SD, length of follow-up, sample size
• Pre-nasal airflow (PNIF) ± SD, post-nasal airflow (PNIF) ± SD, length of follow-up, sample size
• Pre-olfaction (UPSIT) ± SD, post-olfaction (UPSIT) ± SD, length of follow-up, sample size
• Pre-systemic IgE levels ± SD, post-systemic IgE levels ± SD, length of follow-up, sample size
• Pre-spirometric result ± SD, post-spirometric result ± SD, length of follow-up, sample size
• List of adverse events

*PNIF* peak nasal inspiratory flow, *SF-36* 36-Item Short Form Health Survey, *AQLQ* Asthma Quality of Life Questionnaire, *RSOM-31* Rhinosinusitis Outcome Measure 31, *TNSS* Total Nasal Symptom Severity, *SNOT-20* Sinonasal Outcome Test 20, *UPSIT* University of Pennsylvania Smell Identification Test

### Risk of bias assessment

The two reviewers also performed independent risk of bias assessment of included studies using the Cochrane risk of bias tool [23]. Discrepancies were resolved by consensus. Random sequence generation, allocation concealment, blinding of participants and personnel, blinding of outcome assessment, incomplete outcome data, selective reporting, and other sources of bias are the domains of the Cochrane tool. Other sources of potential bias assessed included pharmaceutical company involvement. Each domain was assessed as at a low, unclear, or high risk of bias; these assessments are incorporated in the GRADE judgment of the quality of evidence [24].

### Data analyses

Study characteristics are shown in tables and described narratively. No meta-analysis was carried out because the two included studies used different outcome measures. Where possible, effect estimates for individual studies were reported with mean differences (MDs) and 95 % confidence intervals (CIs), using Review Manager (version 5.3). Where needed, a correlation coefficient of 0.25 was used to impute standard deviations for means used in change from baseline calculations.

### Overall quality of evidence

Two independent reviewers (JQ and JB) used the GRADE framework to judge the overall quality of evidence [25–29]. This assessment involves judgment in the following domains: risk of bias, publication bias, imprecision, inconsistency, and indirectness. GRADE assessments were performed for the body of evidence for each outcome.

## Results

**Fig. 1:**
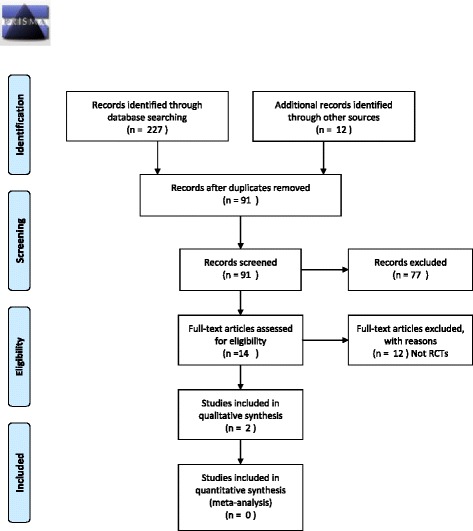
PRISMA 2009 flow diagram. From: Moher D, Liberate A, Tetzlaff J, Altman DG, The PRISMA Group (2009). Preferred Reporting Items for Systematic Reviews and Meta-Analysis: The PRISMA Statement. Plos Med 6(6): e100097. doi:10.1371/journal.pmed100097

A study flow diagram is presented in Fig. 1. Our search identified 239 records, 14 of which remained after removing duplicate entries and excluding non-eligible articles from title and abstract screening. After application of our inclusion criteria by reviewing these potential articles in full-text, two RCTs with a placebo comparison were included. No studies with another therapy as a comparison were identified. No additional ongoing or completed trials were located on ClinicalTrials.gov, ICTRP, and EU Clinical Trials Registry.

### Characteristics of studies

Table 2 represents study characteristics of the two included studies. Both studies assessed anti-IgE monoclonal antibody therapy in adult (>18 years old) CRS patients with serum IgE between 30 and 700 kU/mL. All patients in the treatment group had nasal polyposis and comorbid asthma. The Gevaert et al. [16] study was a multisite RCT conducted at two university hospitals in Belgium, while the Pinto et al. [17] study was a single-site RCT conducted in the USA. Standard drug doses and dosing frequencies used for the treatment of allergic asthma were applied in the included studies. Sample sizes for the studies were small (23 and 14 patients, respectively).

**Table 2:** Study characteristics of the two included studies

Study characteristics		
	Omalizumab is effective in allergic and nonallergic patients with nasal polyps and asthma	A randomized, double-blind, placebo-controlled trial of anti-IgE for chronic rhinosinusitis
First author	Gevaert	Pinto
Publication year	2013	2010
Country	Belgium	USA
Funding source	Ghent University, Flemish Scientific Research Board, Belgian Research Fund, Interuniversity Attraction Poles Program, Global Allergy and Asthma European Network, Novartis	Genentech and McHugh Research Fund, Dennis W. Jahnigen Career Development Award (American Geriatrics Society)
Study design	RCT	RCT
Inclusion criteria	Age ≥18 with CRSwNP, comorbid asthma for >2 years, serum IgE between 30 and 700 kU/mL	Age 18–75, >12 weeks of symptoms with confirmation on CT and nasal endoscopy, serum IgE between 30 and 700 kU/mL
Exclusion criteria	N/A	Weight >150 kg, secondary causes of CRS, contraindications to omalizumab
No. of subjects randomized	24	14
No. of subjects excluded	4	0
No. of subjects withdrawn	1	0
No. of subjects analyzed	Total (*n*) = 23	Total (*n*) = 14
	Men (*n*) = N/A	Men (*n*) = 10
	Women (*n*) = N/A	Women (*n*) = 4
Age	Mean = N/A	Mean = 45.85
	Median = N/A	Median = N/A
	Range = 42–56	Range = N/A
Drug dose	Max dose of 375 mg	0.016 mg/kg per IU total serum IgE/mL
Dosing frequency	Every 2 weeks (eight injections in total); every month (four injections in total)	At enrollment and every 4 weeks for the 6 months duration
Study duration	20 weeks (16 weeks follow-up)	6 months

*RCT* randomized controlled trial, *CRS* chronic rhinosinusitis, *N/A* not available

### Risk of bias assessment

Table 3 outlines the risk of bias assessments by domain for included studies. Overall, studies were deemed at a moderate risk of bias for outcomes.

**Table 3:**
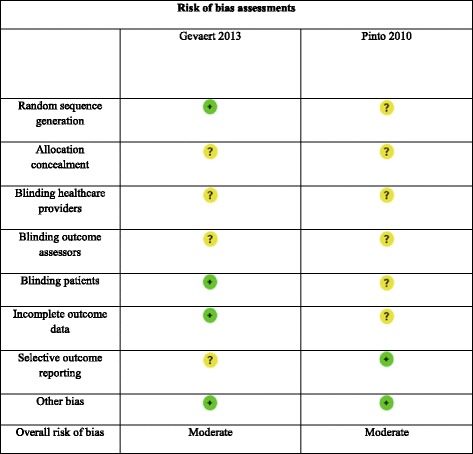
Risk of bias assessments by domain for included studies

There was an adequate random sequence generation in the Gevaert et al. [16] study, but this information was not available in the Pinto et al. [17] study. It was unclear whether allocation concealment took place in either study. We assessed “blinding of participants and personnel” across all outcomes, and in both studies, it was not possible to determine whether personnel (i.e., healthcare providers, outcome assessors) were blinded. In the Gevaert et al. [16] study, participants were not aware of the group (treatment vs. placebo) they had been allocated to. This information was unclear in the Pinto et al. [17] study. Loss-to-follow-up and handling of missing data are important aspects of attrition bias, and both studies were either at low or unclear risk of bias for all outcomes, respectively. In the Gevaert et al. [16] study, one patient was withdrawn and four control patients were excluded; this may have resulted in an overestimate of the treatment effects. There were no dropouts in the Pinto et al. [17] study, and all recruited patients completed the study measures and were analyzed. Studies were also at unclear and low risk for selective reporting bias. Study sponsorship bias (other bias) was not an issue in these studies.

### Effects of intervention

When comparing anti-IgE monoclonal antibody therapy to placebo (Table 4), there was a significant difference in Lund-McKay score [30] in one study. Based on median data, groups did not appear to be different for percent opacification on CT.

**Table 4:** Evidence summary table

Outcome	Study	Study data: anti-IgE vs. placebo	Effect estimate (95 % CI)	Studies (people)	Overall quality of evidence	Comments
Primary outcomes						
Change in CT score						
Lund-McKay Score (change in score from baseline)	Gevaert	Mean (no CI), 4.0 vs. −0.5 (improvement at 16 weeks); *p* = 0.04	See comment	1 (23)	Low	Authors state that scores improved with treatment over control at 16 weeks.
Percent opacification on CT (median change in % inflammation from baseline)	Pinto	Median (IQR), 13.1 % (4.7 to 29.9) vs. 5.9 % (−11.6 to 23.0)	Not estimable	1 (14)	Low	(−) median value means reduced inflammation at 6 months.
Change in clinical polyp score						
Total nasal endoscopic polyp score (change in score from baseline; score 0 to 4, 4 = largest)	Gevaert; Pinto	Mean (SD^a^), −2.67 (2.09) vs. −0.12 (0.99) (smaller polyp size at 16 weeks); see comment	MD −2.55 (−3.81 to −1.29); MD could not be calculated in Pinto et al. trial due to ambiguity in data handling	1 (23); 1 (14)	Low	Pinto et al. trial provided mean data despite nonparametric statistical test. (−) value for MD means greater decrease in polyp size from baseline with anti-IgE monoclonal antibody therapy.
Change in quality of life						
SF-36 (change in score from baseline; physical health, mental health)	Gevaert	Not provided	Not estimable	1 (23)	Low	Authors did not compare change from baseline between groups.
AQLQ (change in score from baseline)	Gevaert	Mean (no CI), 0.81 vs. 0.27 (improvement at 16 weeks)	See comment	1 (23)	Low	Data poorly reported; unclear whether *p* = 0.003 refers to difference in treatment arm from baseline or a comparison from baseline between groups.
RSOM-31 (change in score from baseline)	Gevaert	Not provided	Not estimable	1 (23)	Low	Authors did not compare change from baseline between groups.
TNSS	Pinto	Median, −1 vs. 0; *p* < 0.21	Not estimable	1 (14)	Low	(−) median value means reduced nasal symptoms at 6 months.
SNOT-20 (change in mean from baseline)	Pinto	Mean (SD^a^), 0.98 (1.15) vs. 0.75 (1.76) (improvement at 16 weeks); *p* < 0.60	MD 0.23 (−1.33 to 1.79)	1 (14)	Low	(+) value for MD means greater control of nasal symptoms from baseline with anti-IgE monoclonal antibody therapy.
Secondary outcomes						
Change in cellular inflammation						
Eosinophil count (nasal lavage; median change from baseline)	Pinto	Median (IQR), 2 (−11.75 to 9.25) vs. 9 (−2.75 to 26.5); *p* < 0.47	Not estimable	1 (8)	Low	(+) median value means increased eosinophil count at 6 months.
Change in nasal airflow						
PNIF (median change from baseline)	Pinto	Median (IQR), −0.9 (−20.0 to 40.0) vs. −7.5 (−30.0 to 13.3); *p* < 0.31	Not estimable	1 (12)	Low	(−) median value means reduced nasal airflow at 6 months.
Change in olfaction						
UPSIT	Pinto	Median (IQR), 3 (2 to 14) vs. −4 (−5 to −2); *p* < 0.31	Not estimable	1 (14)	Low	(+) median value means increased smell identification at 6 months.
Adverse events	Gevaert; Pinto	Treatment (4—frontal headache, 3—nasal obstruction, 2—shortness of breath, 1—allergy, 8—common cold, 1—gastroenteritis, 1—shoulder pain, 2—otitis media, 1—left ulnar hypoesthesia, 1—general myalgia) vs. placebo (1—asthma exacerbation, 1—frontal headache, 3—nasal obstruction, 1—shortness of breath, 1—jaundice, 1—acute sinusitis); no adverse events occurred in Pinto et al. trial	See comment	1 (23); 1 (14)	Low	Common cold was the only adverse event to occur more frequently with treatment (*p* = 0.02).
Change in systemic IgE levels						
Not reported in any studies	N/A			N/A		
Change in spirometric results						
Not reported in any studies	N/A			N/A		

*CI* confidence interval, *IQR* interquartile range, *SF-36* 36-Item Short Form Health Survey, *AQLQ* Asthma Quality of Life Questionnaire, *RSOM-31* Rhinosinusitis Outcome Measure 31, *TNSS* Total Nasal Symptom Severity, *SNOT-20* Sinonasal Outcome Test 20, *PNIF* peak nasal inspiratory flow, *UPSIT* University of Pennsylvania Smell Identification Test, *N/A* not available

^a^ *r* = 0.25 used

In the study by Gevaert et al. [16], there was a decrease in clinical polyp score in the treatment group compared to the placebo group at 16 weeks (Table 4). In the study by Pinto et al. [17], a MD could not be calculated because of the ambiguity in data handling.

Several quality of life measures were reported (Table 4). It was unclear whether groups were statistically different for Asthma Quality of Life Questionnaire (AQLQ) because of the poor reporting of the *p* value and absence of data to calculate a CI. Total Nasal Symptom Severity (TNSS) and Sinonasal Outcome Test 20 (SNOT-20) were not statistically significant. Authors did not compare groups for the 36-Item Short Form Health Survey (SF-36) or Rhinosinusitis Outcome Measure-31 (RSOM-31).

A few secondary outcomes were measured. Groups were not different for eosinophil count, peak nasal inspiratory flow (PNIF), and olfactory change, measured with the University of Pennsylvania Smell Identification Test (UPSIT) (Table 4). Neither study reported on measures addressing changes in systemic IgE levels or spirometric results.

No serious adverse events were reported with anti-IgE monoclonal antibody therapy in either trial. The common cold was the only adverse event that occurred more frequently (*p* = 0.02) in patients receiving anti-IgE monoclonal antibody therapy in comparison to placebo (Table 4). With only 37 included patients in this review, however, the sample is inadequate to make global statements of the safety of this therapy in this population. At this time, safety data from the use of anti-IgE monoclonal antibody therapy in allergic asthma alone should be observed.

## Discussion

To date, very few studies have assessed the effectiveness and safety of anti-IgE monoclonal antibody therapy for the management of CRS. As demonstrated in our review, there were only two RCTs comparing anti-IgE monoclonal antibody therapy against placebo and we did not locate any studies evaluating this treatment against other therapies. In this study, GRADE assessments revealed that there is currently a low overall quality of evidence for recommendations regarding anti-IgE monoclonal antibody therapy for the treatment of CRS (see Table 5). Most commonly, each outcome measure was reported by only one study and most outcomes were not statistically significant. Furthermore, the included studies had several important methodological limitations, leading to limited overall quality of evidence. Due to the few included studies with small sample sizes and marked heterogeneity, results were also imprecise and could not be assessed for consistency.

**Table 5:** GRADE assessments for the body of evidence for each outcome and judgment of the overall quality of evidence

Outcome	Follow-up	Risk of bias	Inconsistency	Indirectness	Imprecision	Publication bias	Overall quality of evidence
Primary outcomes							
Change in CT score							
Lund-McKay Score	16 weeks	Moderate limitation	Not relevant	No serious indirectness	Serious imprecision	Unlikely	Low
Percent opacification on CT	6 months	Moderate limitation	Not relevant	No serious indirectness	Serious imprecision	Unlikely	Low
Change in clinical polyp score							
Total nasal endoscopic polyp score	16 weeks; 6 months	Moderate limitation	Not relevant	No serious indirectness	Serious imprecision	Unlikely	Low
Change in quality of life							
SF-36	16 weeks	Moderate limitation	Not relevant	No serious indirectness	Serious imprecision	Unlikely	Low
AQLQ	16 weeks	Moderate limitation	Not relevant	No serious indirectness	Serious imprecision	Unlikely	Low
RSOM-31	16 weeks	Moderate limitation	Not relevant	No serious indirectness	Serious imprecision	Unlikely	Low
TNSS	6 months	Moderate limitation	Not relevant	No serious indirectness	Serious imprecision	Unlikely	Low
SNOT-20	6 months	Moderate limitation	Not relevant	No serious indirectness	Serious imprecision	Unlikely	Low
Secondary outcomes							
Change in cellular inflammation							
Eosinophil count (nasal lavage)	6 months	Moderate limitation	Not relevant	No serious indirectness	Serious imprecision	Unlikely	Low
Change in nasal airflow							
PNIF	6 months	Moderate limitation	Not relevant	No serious indirectness	Serious imprecision	Unlikely	Low
Change in olfaction							
UPSIT	6 months	Moderate limitation	Not relevant	No serious indirectness	Serious imprecision	Unlikely	Low
Adverse events	16 weeks; 6 months	Moderate limitation	Not relevant	No serious indirectness	Serious imprecision	Unlikely	Low

*SF-36* 36-Item Short Form Health Survey, *AQLQ* Asthma Quality of Life Questionnaire, *RSOM-31* Rhinosinusitis Outcome Measure 31, *TNSS* Total Nasal Symptom Severity, *SNOT-20* Sinonasal Outcome Test 20, *PNIF* peak nasal inspiratory flow, *UPSIT* University of Pennsylvania Smell Identification Test

The use of daily saline irrigation, intranasal topical corticosteroids, and/or antibiotics comprise the most commonly utilized agents as part of the CRS treatment protocol. These strategies address the core symptoms by reducing the overall mucosal inflammatory and bacterial burden of the paranasal sinus mucosa. In clinical cases that are poorly responsive to medical therapy, surgical evaluation is often considered. Endoscopic sinus surgery provides an effective means of improving symptom control, and it has been shown to directly improve the health utility values of patients with CRS [8]. However, despite advances in modern treatment strategies, managing patient symptoms of CRS continues to pose challenges for healthcare providers with many patients not responding or only temporarily responding to these therapeutic interventions [31].

In recent years, anti-IgE monoclonal antibody was proposed as a sole or adjuvant therapy to improve outcomes in CRS management paradigms [13–17]. Like asthma, CRS for some patients has been found to be associated with local infiltration of polyclonal IgE—a key mediator in the tissue inflammatory process [10, 32]. *Staphylococcus aureus* enterotoxins are believed to act as superantigens and induce local IgE formation combined with eosinophilic tissue inflammation [32]. Similarly, other causes of local IgE production such as fungal antigen may increase the inflammatory load in CRS [33].

Theoretically, use of anti-IgE monoclonal antibody therapy in CRS makes biologic sense, but there is insufficient research evidence to determine whether it is clinically effective overall or for any subset of disease. If so, it may have important implications for the management paradigm for CRS since local infiltration of IgE and concurrent eosinophilic tissue inflammation are present in up to 80 % of Caucasian patients with CRS [34].

To our knowledge, this systematic review is the first of its kind, setting out to provide a synthesis of the available evidence concerning the use of anti-IgE monoclonal antibody in patients with CRS. Overall, there was little evidence to draw definitive conclusions regarding the effectiveness of anti-IgE monoclonal antibody for the management of CRS. Well-designed RCTs with adequate power are needed to further assess the effectiveness of this treatment in the CRS with nasal polyps and asthma population in the future. In addition, despite several published studies demonstrating safety of anti-IgE monoclonal antibody therapy when used to treat diseases such as allergic asthma and allergic rhinitis [35–37], more research is needed to further characterize its safety profile. Nonetheless, based on current evidence, a trial of anti-IgE monoclonal antibody therapy seems to be a biologically valid option for some patients with CRS who do not respond to conventional treatment regimens.

The greatest limitation of the included studies is the poor reporting of outcome measures, which made it difficult to assess and interpret the reported data. Other items such as standard deviations for means were not reported; authors should adhere to the Consolidated Standards of Reporting Trials (CONSORT) guideline when reporting future trials [38]. In addition, while the Pinto et al. [17] trial included patients who were already refractory to multiple treatments (i.e., population applicability), the Gevaert et al. [16] trial had one patient who withdrew and four placebo patients who were excluded from the study. This affects the assessment of clinical heterogeneity for future meta-analyses, should more studies become available. The variable qualities, definitions, and follow-ups were also important limitations of the included studies. Finally, each of the included studies had a moderate risk of bias, which can lead to an overestimation of the treatment effects and decreases the confidence as to whether the observed effects are the “true” effects of treatment. Future research evaluating the effectiveness and safety of anti-IgE monoclonal antibody therapy should take the aforementioned limitations into consideration. The improved study design will serve as an opportunity to abridge current evidence gaps by identifying the true benefits and adverse events associated with the therapy with an optimal threshold. Future trials should include the core outcomes of a patient CRS symptom score (e.g., SNOT-22), health-related quality of life, olfactory testing, and a clinical polyp score for CRS with polyp patients.

A limitation of this systematic review is that we could have potentially missed otherwise eligible studies in other languages as our inclusion criteria were limited to studies reported in the English language. However, based on the small number of included studies, we feel that the likelihood of studies existing in other languages is quite low. In addition, our review only had one reviewer involved in the screening and eligibility assessment of relevant articles. Finally, we did not address anti-IgE monoclonal antibody as an adjuvant therapy. In the future, it may be beneficial to assess whether there is greater effectiveness in using anti-IgE monoclonal antibody alongside the prevailing therapeutic interventions.

## Conclusions

There is currently insufficient evidence to determine whether anti-IgE monoclonal antibody therapy is more effective or safer than placebo for the treatment of CRS. A sufficiently powered, high-quality trial is needed to further assess its effectiveness in this population. Determining its effectiveness and safety may have important implications for the management paradigm of some patients with CRS who do not respond to conventional treatment regimens.

## Additional file

Additional file 1:
**PRISMA 2009 Checklist.** (DOC 62 kb)

## Appendix 1

### Medline search (Ovid)

Search strategy:

1. exp Antibodies, Monoclonal/tu [Therapeutic Use]
2. exp Antibodies, Anti-Idiotypic/tu [Therapeutic Use]
3. exp Antibodies, Monoclonal, Humanized/tu [Therapeutic Use]
4. anti IgE.mp.
5. (anti adj1 IgE).mp. [mp = title, abstract, original title, name of substance word, subject heading word, keyword heading word, protocol supplementary concept word, rare disease supplementary concept word, unique identifier]
6. anti IgE therapy.mp.
7. omalizumab.mp.
8. 1 or 2 or 3 or 4 or 5 or 6 or 7
9. exp Sinusitis/
10. sinusitides.tw.
11. 9 or 10
12. exp Rhinitis/
13. (catarrh* adj2 nasal).tw.
14. (rhinitis adj2 nonseasonal).tw.
15. 12 or 13 or 14
16. 11 and 15
17. exp Nasal Polyps/
18. polyp* nasal.tw.
19. nasal polyp*.tw.
20. 17 or 18 or 19
21. 16 or 20
22. 8 and 21

## Abbreviations

AQLQ: Asthma Quality of Life Questionnaire
CI: confidence interval
CRS: chronic rhinosinusitis
CT: computed tomography
CONSORT: Consolidated Standards of Reporting Trials
ICTRP: International Clinical Trials Registry Platform
MD: mean difference
PRISMA: Preferred Reporting Items for Systematic Reviews and Meta-analyses
PNIF: peak nasal inspiratory flow
RCT: randomized controlled trial
RSOM-31: Rhinosinusitis Outcome Measure 31
SF-36: 36-Item Short Form Health Survey
SNOT-20: Sinonasal Outcome Test 20
TNSS: Total Nasal Symptom Severity
UPSIT: University of Pennsylvania Smell Identification Test
